# Synthetic engineering of a new biocatalyst encapsulating [NiFe]-hydrogenases for enhanced hydrogen production†

**DOI:** 10.1039/d2tb02781j

**Published:** 2023-02-20

**Authors:** Qiuyao Jiang, Tianpei Li, Jing Yang, Catherine M. Aitchison, Jiafeng Huang, Yu Chen, Fang Huang, Qiang Wang, Andrew I. Cooper, Lu-Ning Liu

**Affiliations:** a Institute of Systems, Molecular and Integrative Biology, University of Liverpool Liverpool L69 7ZB UK luning.liu@liverpool.ac.uk; b State Key Laboratory of Crop Stress Adaptation and Improvement, School of Life Sciences, Henan University Kaifeng 475004 China; c Materials Innovation Factory and Department of Chemistry, University of Liverpool Liverpool L7 3NY UK; d College of Marine Life Sciences, and Frontiers Science Center for Deep Ocean Multispheres and Earth System, Ocean University of China Qingdao 266003 China

## Abstract

Hydrogenases are microbial metalloenzymes capable of catalyzing the reversible interconversion between molecular hydrogen and protons with high efficiency, and have great potential in the development of new electrocatalysts for renewable fuel production. Here, we engineered the intact proteinaceous shell of the carboxysome, a self-assembling protein organelle for CO_2_ fixation in cyanobacteria and proteobacteria, and sequestered heterologously produced [NiFe]-hydrogenases into the carboxysome shell. The protein-based hybrid catalyst produced in *E. coli* shows substantially improved hydrogen production under both aerobic and anaerobic conditions and enhanced material and functional robustness, compared to unencapsulated [NiFe]-hydrogenases. The catalytically functional nanoreactor as well as the self-assembling and encapsulation strategies provide a framework for engineering new bioinspired electrocatalysts to improve the sustainable production of fuels and chemicals in biotechnological and chemical applications.

## Abstract 2

By incorporating [NiFe]-hydrogenases into a proteinaceous carboxysome shell, we generate a novel biocatalyst that has improved production of clean hydrogen, oxygen tolerance, and thermostability, highlighting its great potential in biotechnological applications.

## Graphical abstract

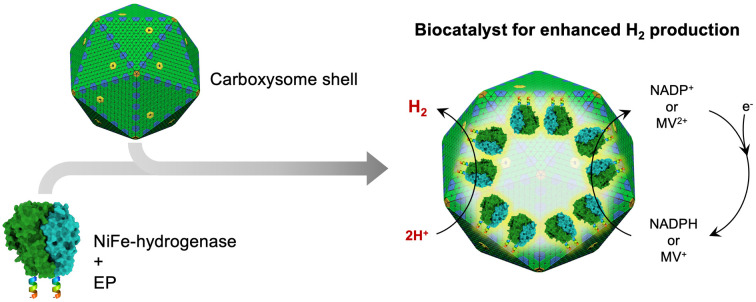

## Introduction

Rapid human development and increasing energy demand have had a massive impact on the climate, and the search for new sustainable energy supplies to mitigate climate change has become an important and urgent global challenge. Compared to traditional fossil fuels, hydrogen has advantages as a green energy storage medium when generated renewably from water, owing to its higher energy density (∼120 kJ g^−1^, 3 times more than gasoline or diesel) and clean combustion for a low-carbon future.^1,2^ As such, developing novel catalysts for hydrogen production from water has become a target in chemistry and biology for researchers and engineers to underpin hydrogen economy and address the social challenges.

Many microorganisms have evolved special metalloenzymes, namely hydrogenases, to produce molecular hydrogen from protons and electrons using inexpensive and abundant metals.^3^ Three major phylogenetical classes of hydrogenases have been identified based on their active site metals: [Fe]-hydrogenases, [FeFe]-hydrogenases and [NiFe]-hydrogenases.^4^ Given their superb electrocatalytic activities, hydrogenases provide great promise for constructing new hydrogen-evolution catalysts. However, these enzymes have intrinsic limitations, such as high sensitivity to oxygen and pH and poor thermostability.^5^ To address these issues and enhance hydrogen production, many strategies have been implemented, including integrating hydrogenases into immobilization matrixes^6,7^ or scaffolding materials.^8–11^

Intriguingly, the encapsulation principle has been exploited by a variety of microbes in nature. A paradigm of the encapsulating systems is a self-assembling proteinaceous organelle, known as the carboxysome, an anabolic bacterial microcompartment (BMC) found in all cyanobacteria and many proteobacteria.^12–18^ Carboxysomes encapsulate the key CO_2_-fixing enzyme, ribulose-1,5-bisphosphate carboxylase oxygenase (Rubisco), using a polyhedral protein shell; unlike virus capsids that do not possess permeability, the carboxysome shell is selectively permeable to HCO_3_^−^ and protons while diminishing O_2_ entry and CO_2_ leakage.^19–23^ This elaborate bacterial organelle provides elevated levels of CO_2_ around Rubisco to enhance carbon fixation on the global scale and represents an attractive engineering objective in synthetic biology.^24–28^ Recent studies have shown that direct encapsulation of O_2_-sensitive [FeFe]-hydrogenases into the cavity of a synthetic α-carboxysome shell resulted in improved hydrogen production and O_2_ tolerance of the hybrid biocatalyst, taking advantage of the unique α-carboxysome shell permeability and confinement of cargo enzymes.^29^

In contrast, [NiFe]-hydrogenases are relatively O_2_ tolerant and can catalyze H_2_ oxidation in the presence of O_2_, and have high H_2_-evolution activities under anaerobic conditions.^30–32^ While [NiFe]-hydrogenases are promising candidates as functional catalysts in biotechnological applications,^33^ optimization of [NiFe]-hydrogenases and their surrounding microenvironment is still needed for improved catalytic performance and robustness.

Here, we generate a new hybrid catalyst by sequestering [NiFe]-hydrogenase-1 from *Escherichia coli* (*Ec*Hyd-1) within the recombinant shell of the α-carboxysome from a chemoautotroph *Halothiobacillus neapolitanus*. This catalyst exhibits a substantial increase in hydrogen evolution under aerobic and anaerobic conditions compared to unencapsulated *Ec*Hyd-1 hydrogenases. We also demonstrate that shell encapsulation results in improved oxygen tolerance and thermostability of functional [NiFe]-hydrogenase cargos in hydrogen production. Our study represents a step towards repurposing self-assembling bio-systems to develop sustainable biocatalysis in biotechnological applications.

## Experimental

### Construction of recombinant [NiFe]-hydrogenase-1

Genes encoding HyaA and HyaB were amplified from the *E. coli* BL21(DE3) genome *via* using CloneAmp HiFi polymerase (Takara, Japan) (Table S1, ESI†). The C-terminus of full-length CsoS2 served as an encapsulation peptide (EP) and was amplified from the pHnCBS1D plasmid (Addgene, US). The EP sequence was fused to the 3′-end of *hyaB* followed by ligation to the pCDFDuet-1 vector linearized by EcoRI and HindIII to produce the pCDF-*hyaB*-EP vector. The *hyaA* gene with the C-terminus fused with the CsoS2 C-terminus was ligated to the pCDF-*hyaB*-EP vector linearized by NdeI and XhoI to generate pCDF-*hyaAB*-EP. The resulting PCR products and linearized vectors were purified by using a DNA gel extraction kit (New England BioLabs, UK) following the standard manufacture protocol. All vectors were verified by PCR and DNA sequencing (IDT, US) and transformed into *E. coli* BL21(DE3) competent cells.

### Expression of recombinant [NiFe]-hydrogenase-1 and α-carboxysome shells

For the expression of [NiFe]-hydrogenases, *E. coli* strains containing HyaAB-EP plasmids were grown in LB medium containing 50 μg mL^−1^ spectinomycin, 0.03 mM (final) ferric ammonium citrate and 0.03 mM (final) nickel chloride monohydrate, and expression was induced by adding 0.05 mM isopropyl β-d-thiogalactopyranoside (IPTG) at OD_600_ = 0.6. For the co-expression of α-carboxysome shells and mature [NiFe]-hydrogenases, the empty shell expression plasmid was generated as reported,^29^ including the *csoS*2, *csoS*4*AB*, *csoS*1*ABC* and *csoS1D* genes. The HyaAB-EP plasmid was transformed into *E. coli* BL21(DE3) competent cells containing Shell. Cells were grown at 37 °C in LB medium supplemented with 50 μg mL^−1^ spectinomycin, 100 μg mL^−1^ ampicillin, 0.03 mM (final) ferric ammonium citrate and 0.03 mM (final) nickel chloride monohydrate. Expression of HyaAB-EP was induced at OD_600_ = 0.6 by adding 0.05 mM IPTG at 25 °C. L-arabinose was added at a final concentration of 1 mM 4 hours after IPTG induction to initiate shell expression.

### Purification of recombinant HyaAB-Shell assemblies

Cells were harvested by centrifugation at 5000 × *g* for 10 minutes at 4 °C. The pellets were washed with TMB buffer (10 mM Tris-HCl pH 8.0, 10 mM MgCl_2_, 20 mM NaHCO_3_) twice and resuspended in 20 mL of TMB buffer. The resuspended cells with 10% (v/v) CelLytic B cell Lysis reagent (Sigma-Aldrich) were lysed by French press (STANSTED FPG12800 pressure cell homogenizer) following the standard recommend process. Cell debris was removed by initial centrifugation at 10 000 × *g* for 10 minutes at 4 °C. After centrifugation at 50 000 × *g* for 30 minutes at 4 °C, the supernatant was discarded and the pellet was gently resuspended in 2 mL TMB buffer using a soft brush. A 2 minute spin at 4 °C was performed to remove the insoluble fraction. The soluble pellet fraction was applied onto a step sucrose gradient (10–50%, w/v, solubilized in TMB buffer) for ultracentrifugation at 105 000 × *g* for 35 minutes. Sucrose fractions were separately collected and stored at 4 °C for further analysis.

### SDS-PAGE and immunoblot analysis

Protein samples were prepared and then mixed with 4× sodium dodecyl-sulfate polyacrylamide gel electrophoresis (SDS-PAGE) sample loading buffer (250 mM Tris pH 6.8, 8% SDS, 0.2% bromophenol blue, 40% glycerol, 20% mercaptoethanol). After 95 °C heating for 10 minutes, samples were centrifuged at 12 000 × *g* for 2 minutes and then loaded on 15% SDS-PAGE gels to analyze their composition. About 75 μg proteins were loaded in each SDS-PAGE gel well. 3 μL of unstained protein ladder (10–250 kDa from NEB) was loaded as a marker. SDS-PAGE was run at 200 V for 45 minutes with SDS running buffer (25 mM Tris, 192 mM glycine and 1% SDS). The gels were stained in Coomassie blue stain buffer (0.25% Coomassie Brilliant Blue R-250, 20% methanol, 10% acetic acid) and destained by destaining buffer (20% methanol, 10% acetic acid).

Proteins (30 μg) were loaded onto 15% SDS-PAGE gels and then transferred to PVDF (polyvinylidene difluoride) membranes (Bio-Rad). Immunoblot analysis was performed using primary mouse monoclonal anti-Histag (Life Technologies, 69-74-9, UK), primary rabbit polyclonal anti-CsoS1A/B/C (Agrisera, AS142760, dilution 1 : 5000, US), as well as horseradish peroxidase-conjugated goat anti-mouse IgG secondary antibody (Agrisera, AS111772 dilution 1 : 10 000, US) and anti-rabbit IgG secondary antibody (Agrisera, AS09602, dilution 1 : 10 000, US). Then, the membranes were washed with TBS buffer (10 mM Tris-HCl pH 7.4, 150 mM NaCl) and TBST buffer (10 mM Tris-HCl pH 7.4, 150 mM NaCl, 0.1% Tween-20). Immunoblot signals were developed by using a Bio-Rad chemiluminescence kit and images were recorded *via* ImageQuant LAS 4000 software version 1.2.1.119.

Free HyaAB-EP were isolated through a HisTrap HP column (Cytiva, UK), and were loaded on a 15%(v/v) SDS-PAGE gel. HyaB-EP proteins were extracted from SDS-PAGE gels for protein quantification as the reference for the quantification of HyaAB-EP content in the unencapsulated and encapsulated forms, according to previous studies.^29^ The purified HyaB-EP at various protein concentrations were loaded onto SDS-PAGE gels for immunoblot analysis, which allowed us to generate a standard curve according to the linear relationship between the HyaB-EP protein band intensities and the amount of loaded HyaB-EP proteins. Protein quantification analysis was performed by using ImageJ software (version 1.52 h). For each experiment, at least three biological replicates were examined.

### Transmission electron microscopy (TEM)

Isolated protein samples (1–2 mg mL^−1^ total protein) were stained on carbon grids (Carbon Films on 300-mesh Grids Copper, Agar Scientific) for 40 seconds, followed by staining with 2% (v/v) uranyl acetate (Sigma-Aldrich). The carbon grids were then washed with distilled water and then dried using 0.2 μm filter paper. Images were recorded using an FEI Tecnai G2 Spirit Bio TWIN transmission electron microscope equipped with a Gatan Rio 16 camera. ImageJ was used for the image analysis. Statistical analysis was calculated by using Student's *t*-test.

### Dynamic light scattering (DLS) analysis

Briefly, 1 mL (5–10 mg mL^−1^ total protein) of samples were analyzed by dynamic light scattering (Malvern DLS ZetaSizer) to measure the size distribution and average size of the particles. For each experiment, at least three biological replicates were examined.

### *In vivo* dissolved oxygen (DO) measurement

A polarographic DO probe (New BrunswickTM BioFlo/CellGen 115 Fermentor, Eppendorf) was used to measure dissolved oxygen (DO) in strains expressing free HyaAB-EP, HyaAB-Shell for *in vivo* activity assays. Oxygen-saturated LB medium (100% DO) and sodium dithionite (DT)-treated LB medium (0% DO) were used to calibrate the polarized polarographic DO probe, respectively. The DO levels of strains expressing free HyaAB-EP and HyaAB-Shell were determined before adding IPTG and every four hours after adding IPTG and l-arabinose. For each experiment, at least three biological replicates were examined.

### *In vivo* H_2_-evolution assay

Strains expressing free HyaAB, free HyaAB-EP and HyaAB-Shell were aerobically grown at 37 °C in a 200 mL flask with 50 mL LB medium supplemented with 0.03 mM (final) ferric ammonium citrate and 0.03 mM (final) nickel chloride monohydrate, 50 μg mL^−1^ spectinomycin, and/or 100 μg mL^−1^ ampicillin. At OD_600_ = 0.6, 30 mL culture was transferred to a 50 mL falcon tube with sealed rubber closure (Sigma-Aldrich) and was degassed by 100% nitrogen for 5 mins before the addition of IPTG, l-cysteine, and sodium fumarate for anaerobic treatment. For aerobic treatment, 30 mL culture was transferred to a falcon tube with a sealed rubber closure without a N_2_ degassing process. 1 mM l-arabinose was added 4 hours after the addition of 0.05 mM IPTG for strains containing HyaAB-Shell. Cells were grown at 25 °C for 16 hours with constant shaking following induction. 1 mL gas samples were taken with a gas-tight syringe and a sample loop was flushed (100 μL) with the sample. The sample loop was then switched and run on a Bruker 450-GC gas chromatograph. The system was equipped with a molecular sieve 13 × 60–80 mesh 1.5 m × 1/8 in. × 2 mm ss column at 50 °C with an argon flow of 40.0 mL min^−1^. Hydrogen was detected with a thermal conductivity detector referencing against standard gas with a known concentration of hydrogen. For each experiment, at least three biological replicates were examined.

### *In vitro* H_2_-evolution assay

Cells were grown aerobically at 37 °C in a 2 L flask containing 800 mL LB medium with the corresponding antibiotics, 0.03 mM (final) ferric ammonium citrate and 0.03 mM (final) nickel chloride monohydrate, 50 μg mL^−1^ spectinomycin, and/or 100 μg mL^−1^ ampicillin. At OD_600_ = 0.6, the culture was degassed by 100% N_2_ for 30 min, and then HyaAB-EP expression was induced by 0.05 mM IPTG. For strains containing HyaAB-Shell, 0.05 mM IPTG was added for HyaAB-EP expression. After a 4 hour induction, 1 mM (final) l-arabinose was added for shell expression and the cells were then grown at 25 °C with constant shaking.

The whole purification process was performed under anaerobic conditions, followed by *in vitro* hydrogenase H_2_-evolution activity assays. For *in vitro* hydrogenase kinetics assays, the protein amount of HyaAB in the samples containing free HyaAB or HyaAB-Shell were quantified by immunoblot using purified HyaB as the reference (Fig. S6, ESI†). Then, samples (0.5 mL, ∼10 mg mL^−1^) containing equal amounts of HyaB in TMB buffer were mixed with 100% nitrogen-degassed methyl viologen (MV^+^) (0–200 mM, final) and DT (500 mM, final) in sealed serum vials (Agilent Technologies) inside anaerobic glove bags. The vials were incubated at 37 °C for 16 hours with constant shaking and were then assayed using a Bruker 450-GC gas chromatograph for hydrogen production. Hydrogenase activity at a range of MV concentrations was plotted and fitted using a standard Michaelis–Menten model. For time series measurement, hydrogen evolution of HyaAB-Shell with 50 mM MV and 500 mM DT was measured every 20 minutes in comparison with free HyaAB-EP as a control. For each experiment, at least three biological replicates were examined.

### Oxygen exposure and heat treatment

0.5 mL free HyaAB-EP and HyaAB-Shell samples (∼10 mg mL^−1^) isolated under anaerobic conditions were exposed to the air at 4 °C for 24 hours, respectively. Then, the samples were transferred into 10 mL sealed serum vials and were degassed by pure nitrogen for 5 minutes. The samples were incubated with 2 mL nitrogen-degassed MV (50 mM in TMB buffer, final) and 0.5 mL DT (500 mM in TMB buffer, final) at 37 °C for 20 minutes and were then subjected to hydrogen evolution assays.

For heat treatment, the protein samples (∼10 mg mL^−1^) were heat treated at 65 °C for 20 minutes under anaerobic conditions, followed by incubation with 2 mL nitrogen-degassed MV (50 mM in TMB buffer, final) and 0.5 mL DT (500 mM in TMB buffer, final) in 10 mL sealed serum vials at 37 °C for 20 minutes. The samples were then subjected to hydrogen evolution assays. For each experiment, at least three biological replicates were examined.

## Results and discussion

### Generation of [NiFe]-hydrogenase-encapsulated α-carboxysome shells

*Ec*Hyd-1 is encoded by a gene operon of six genes (*hyaABCDEF*).^34^ HyaA is a small core subunit containing three [Fe–S] clusters and is involved in electron transfer, whereas HyaB is a large core subunit consisting of a bimetallic active site.^35–39^ HyaC is an integral membrane cytochrome *b* subunit that is responsible for anchoring *Ec*Hyd-1 to the cytoplasmic membrane.^40^ HyaD, HyaE and HyaF were assumed to be involved in hydrogenase biosynthesis and maturation,^3,37,41,42^ but their exact roles are still poorly understood. Previous studies have shown that HyaAB-only assemblies exhibit H_2_-producing activities and there was no significant difference in H_2_ production between *E. coli* cells expressing *hyaABCDEF* and *hyaAB* only.^43,44^

**Fig. 1:**
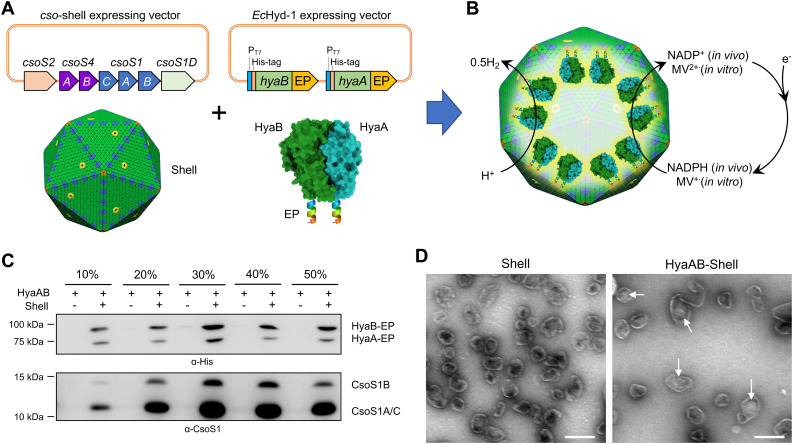
Generation of [NiFe]-hydrogenase-encapsulating catalyst based on the α-carboxysome shell. (A) Genetic organization of the vectors producing synthetic α-carboxysome shells and [NiFe]-hydrogenases (HyaAB). The C-termini of HyaA and HyaB were fused with the CsoS2 C-terminus as the encapsulation peptide (EP) to ensure cargo encapsulation. PDB ID for HyaAB: 3USC. (B) Schematic of the HyaAB-Shell hybrid catalyst comprising the α-carboxysome shell and HyaAB-EP. Hydrogen production by the nanoreactor systems was evaluated by using endogenous NADPH in cells as the electron donor for *in vivo* assays and methyl viologen (MV^+^), which was chemically reduced by sodium dithionite (DT), as the electron donor for *in vitro* tests. Note that the copy number of [NiFe]-hydrogenases depicted in the model does not represent the real content of [NiFe]-hydrogenases encapsulated within the hybrid catalyst, which remains speculative. (C) Immunoblot analysis confirmed the presence of HyaAB and shell proteins in the HyaAB-Shell sample purified from sucrose gradient ultracentrifugation. (D) Electron microscopy (EM) images of isolated empty shells and HyaAB-Shell from 20% sucrose fractions. The potential cargo enzymes in the lumen of the shells are indicated by arrows. Scale bar: 200 nm.

To create an α-carboxysome shell encapsulating *Ec*Hyd-1, we first generated a *hyaAB*-expressing vector (*hyaAB*-EP), which can express HyaA and HyaB with both of their C-termini fused with the C-terminus of CsoS2 (Fig. 1A and Fig. S1, ESI†). In our previous studies, we demonstrated that the C-terminus of CsoS2 could serve as an encapsulation peptide (EP) to bind with shell proteins and efficiently recruit non-native cargos, for example fluorescent proteins, algal [FeFe]-hydrogenases, and ferredoxin-NADP^+^ reductase, into the α-carboxysome shell; without the fusion with the CsoS2 C-terminus, foreign cargo proteins could not be incorporated within the shells.^19,29^ The *hya*AB-EP vector was then transformed into the *E. coli* BL21(DE3) cells comprising an α-carboxysome shell-expressing vector, which can express α-carboxysome shell proteins (CsoS4A, CsoS4B, CsoS1A, CsoS1B, CsoS1C, CsoS1D, Fig. 1A) resulting in the formation of empty α-carboxysome shells.^19,29^ Expression of the *hya*AB-EP plasmid was induced by adding IPTG for 4 hours before arabinose-induced shell expression, to ensure heterologous production of functional [NiFe]-hydrogenases prior to shell encapsulation (Fig. 1B).

To verify the encapsulation of HyaAB into α-carboxysome shells, we purified recombinant shells containing HyaAB-EP (HyaAB-shell) from *E. coli* using centrifugation and sucrose gradient ultracentrifugation. The components of HyaAB-Shell, including shell proteins and HyaAB-EP, were detected in the pellet after 50 000 × *g* centrifugation, whereas unencapsulated HyaAB-EP was only present in the supernatant, indicating the formation of HyaAB-Shell assemblies (Fig. S2, ESI†). After sucrose gradient ultracentrifugation, individual sucrose fractions were collected. Immunoblot analysis revealed that HyaA-EP, HyaB-EP, and CsoS1 shell proteins were detectable in 10–50% sucrose fractions, predominantly enriched in the 20% and 30% sucrose fractions (Fig. 1C). In contrast, unencapsulated HyaAB-EP in the absence of shells were not detected in 10–50% sucrose fractions (Fig. 1C), confirming the EP-mediated encapsulation of HyaAB into the shells. Along with previous evidence revealing that CsoS2 C-terminus could drive the encapsulation of [FeFe]-hydrogenases with cofactors and fluorescence proteins,^19,29^ we provide an efficient encapsulation approach using the CsoS2 C-terminus as an EP to encase foreign cargos within the α-carboxysome shells in synthetic biology.

The HyaAB-shell assemblies at the 20% sucrose fraction were further examined using negative-staining electron microscopy (EM) (Fig. 1D). HyaAB-shells exhibit a polyhedral structure with a mean diameter of ∼105 nm as examined by dynamic light scattering (DLS) (Fig. S3A, ESI†), comparable to native α-carboxysomes from *H. neapolitanus*, synthetic α-carboxysomes and empty α-carboxysome shells.^27–29^ Moreover, the shell size gradually increased from 20% to 30% sucrose fractions (Fig. S3B, ESI†).

### Shell encapsulation stimulates hydrogen evolution of [NiFe]-hydrogenase

The HyaB subunit of *Ec*Hyd-1 carries the [NiFe] active site, whereas HyaA contains [Fe–S] clusters that transfer electrons to the active site of HyaB.^40,45,46^ Recent studies revealed that HyaB only encased in the bacteriophage P22 capsid was unable to produce high-yield H_2_,^10^ confirming the importance of HyaA in hydrogen production. The presence of both HyaA and HyaB provides a promise for producing catalytically active *Ec*Hyd-1 within the α-carboxysome shells (Fig. 1C).

**Fig. 2:**
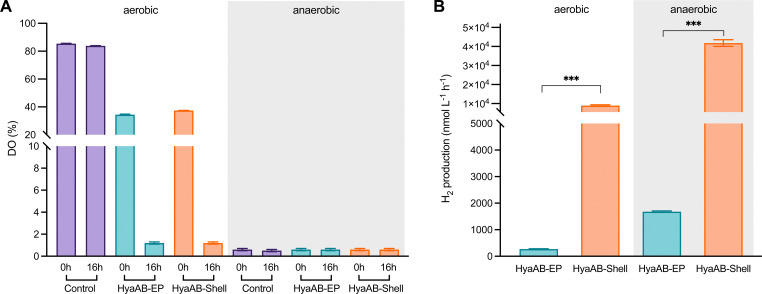
*In vivo* hydrogen production of *E. coli* cells expressing HyaAB-Shell and free HyaAB-EP. (A) The levels of dissolved oxygen (DO) in LB medium (control), LB medium containing the *E. coli* expressing HyaAB-EP or Shells-HyaAB-EP under aerobic and anaerobic conditions during 16-hour induction. Water-saturated DO was set as 100%. The data are presented as the average of nine (or three for control) DO measurements of nine (or three) distinct cell cultures. (B) *In vivo* hydrogenase activity assays. Hydrogen production of *E. coli* cells expressing free HyaAB-EP or HyaAB-Shell grown under aerobic (left) and anaerobic (right) conditions was measured using gas chromatography, normalized by inducing 30 mL cultures after 16 hours. ***, *p* < 0.001, two-tailed unpaired *t*-test. Error bars represent the standard deviation of the mean of three biological replicates.

The H_2_-evolution activities of the *E. coli* cells expressing HyaAB-Shell and free HyaAB-EP grown under aerobic or anaerobic conditions were assayed, using endogenous NADPH in *E. coli* as the electron source (Fig. 1B). First, we measured the levels of dissolved oxygen (DO) in the two different types of cell cultures under aerobic and anaerobic conditions (Fig. 2A). After 16 hour cell culturing in falcon tubes under aerobic conditions, DO dropped from 37.2% to 1.2% (*n* = 3) for the HyaAB-Shell and from 38.4% to 1.2% (*n* = 3) for free HyaAB-EP, demonstrating that the two constructs consumed a large amount of oxygen. The final DO levels of all cell types under aerobic conditions were higher than those under anaerobic conditions (remaining constant at ∼0.6%, Fig. 2A).

*In vivo* H_2_-evolution assays at pH 7 using gas chromatography revealed that the H_2_-production rate of *E. coli* cells expressing HyaAB-Shell under anaerobic conditions was 41 777.5 ± 1753.4 nmol L^−1^ h^−1^ (*n* = 3), more than 23-fold greater than that of cells expressing unencapsulated HyaAB-EP (1,780.1 ± 197.9 nmol L^−1^ h^−1^, *n* = 3) (Fig. 2B and Fig. S4, ESI†). Intriguingly, under aerobic conditions, the H_2_-production rate of cells producing HyaAB-Shell (8,937.4 ± 414.5 nmol L^−1^ h^−1^, *n* = 3) was ∼33-fold higher than that of cells expressing free HyaAB-EP (269.2 ± 15.2 nmol L^−1^ h^−1^, *n* = 3). Since there was no drastic difference in oxygen consumption between the two cell lines (Fig. 2A), the possibility that the different hydrogenase activities of the two cell constructs were ascribed to their distinct oxygen-consumption capacities can be excluded. Moreover, the hydrogen-production activity of *E. coli* BL21(DE3) expressing only endogenous [NiFe]-hydrogenases accounts for <20% of that of *E. coli* BL21(DE3) expressing free HyaAB-EP (Fig. S5, ESI†), suggesting that endogenous [NiFe]-hydrogenases had no significant effect on the overall hydrogen-production performance of *E. coli* BL21(DE3) producing unencapsulated HyaAB-EP and HyaAB-Shell. Collectively, our results indicate that hydrogen production of *Ec*Hyd-1 is greatly enhanced by direct encapsulation of the α-carboxysome shell, consistent with the previous observation of the α-carboxysome shell-based nanoreactor that sequesters [FeFe]-hydrogenases.^29^ It is likely that the lower O_2_ levels and enriched cargo enzyme concentrations developed within the shell provided a catalytically favorable microenvironment for *Ec*Hyd-1 hydrogenases to catalyze the reversible reduction of protons into hydrogen. In addition, cells producing [NiFe]-hydrogenase-encapsulated shells have a much greater hydrogen-production capacity than cells synthesizing [FeFe]-hydrogenase-encapsulated shells^29^ under both aerobic and anaerobic conditions, highlighting the remarkable catalytic performance of the [NiFe]-hydrogenase-packaged nanoreactor.

**Fig. 3:**
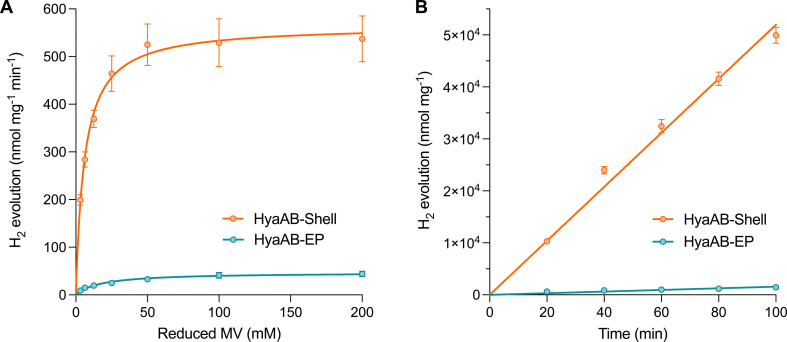
*In vitro* hydrogen production of the HyaAB-Shell catalyst. (A) Hydrogen production activities of anaerobically purified free HyaAB-EP and HyaAB-Shell at pH 8 as a function of different concentrations of nitrogen-degassed MV as the electron mediator reduced by DT, fitted with Michaelis–Menten kinetics. (B) Kinetic hydrogen production (measured at pH 8) of anaerobically isolated free HyaAB-EP and HyaAB-Shell using MV^+^ reduced by 500 mM DT as the electron mediator. Error bars show the standard deviation of the mean of three biological replicates.

We also performed *in vitro* H_2_-evolution assays of free HyaAB-EP and HyaAB-Shell isolated under anaerobic conditions using gas chromatography, based on quantification of the HyaB-EP content (Fig. S6, ESI†). Nitrogen-degassed methyl viologen (MV^+^) with varying concentrations was used as the electron donor reduced by DT in the assays (Fig. 1B). After 16 hour reaction at 37 °C under anaerobic conditions, the maximum H_2_-evolution rate of HyaAB-Shell at pH 8 was 543.4 ± 67.7 nmol H_2_ mg^−1^ min^−1^ (*n* = 3), ∼12-fold greater than that of free HyaAB-EP (47.3 ± 1.6 nmol H_2_ mg^−1^ min^−1^, *n* = 3) (Fig. 3A). Moreover, the amount of H_2_ produced by HyaAB-Shell increased linearly as a function of time at 50 mM MV, notably greater than that produced by free HyaAB-EP, signifying the electrocatalytic capabilities of HyaAB-Shell for H_2_ evolution (Fig. 3B). These results demonstrate explicitly the enhanced H_2_-production performance of [NiFe]-hydrogenases within the α-carboxysome shell-based catalyst, predominantly taking advantage of the reduced O_2_ levels and enriched cargo concentrations by shell encapsulation.

### Shell encapsulation enhances oxygen tolerance and thermostability of [NiFe]-hydrogenase

Compared to the highly O_2_-sensitive [FeFe]-hydrogenase, [NiFe]-hydrogenase is an O_2_-tolerant catalyst that retains certain hydrogen turnover activities in the presence of O_2_.^47–51^ Our *in vivo* assays also revealed that the H_2_-producing rate of free [NiFe]-hydrogenases under anaerobic conditions was ∼6.6-fold greater than that under aerobic conditions (Fig. 2B). Since O_2_ is the only varying factor in this experiment, our results highlight the dominant effect of O_2_ inactivation on tuning the catalytic activity of free [NiFe]-hydrogenases and the necessity of protecting [NiFe]-hydrogenases from O_2_ damage.

**Fig. 4:**
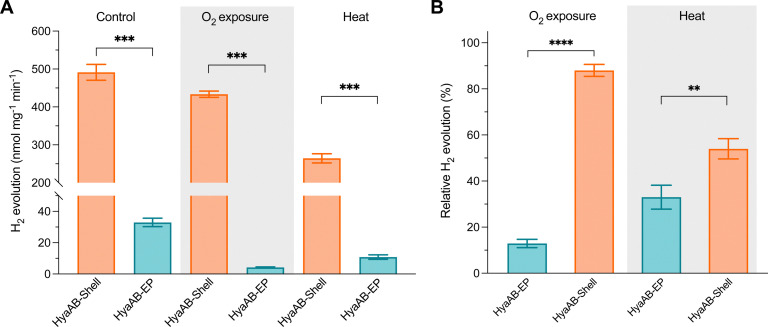
The HyaAB-Shell catalyst shows enhanced oxygen tolerance and thermostability. (A) Hydrogen production activities of isolated free HyaAB-EP and HyaAB-Shell before and after O_2_ exposure and 65 °C treatment at 50 mM MV. *p* = 0.0006 for control; *p* = 0.0001 for O_2_ exposure, *p* = 0.0006 for heat treatment (two-tailed unpaired *t*-test). (B) Relative hydrogen production activities of anaerobically purified free HyaAB-EP and HyaAB-Shell after O_2_ exposure for 24 hours at 4 °C (**** p < 0.0001, two-tailed unpaired *t*-test) and for 20min heat treatment under anaerobic conditions at 65 °C (** *p* = 0.0072, two-tailed unpaired *t*-test), as a relative percentage of total activities measured under anaerobic conditions in (A). Values represent the mean of three independent biological replicates and standard deviation.

To examine the O_2_-tolerance of HyaAB-Shell, we exposed purified HyaAB-Shell and unpackaged HyaAB-EP to ambient air for 24 hours, followed by degassing and H_2_-evolution assays using 50 mM nitrogen-degassed MV^+^ reduced by DT as the electron donor (Fig. 4). After O_2_ exposure, free HyaAB-EP had only ∼13% (4.2 ± 0.3 nmol H_2_ mg^−1^ min^−1^, *n* = 3) of the H_2_-evolution activity measured before O_2_ exposure (32.9 ± 2.7 nmol H_2_ mg^−1^ min^−1^, *n* = 3). By comparison, HyaAB-Shell retained ∼88% (433.6 ± 8.3 nmol H_2_ mg^−1^ min^−1^, *n* = 3) of the H_2_-evolution activity before O_2_ exposure (491.4 ± 21.0 nmol H_2_ mg^−1^ min^−1^, *n* = 3) at pH 8 (Fig. 4A and B). These results indicate explicitly that the protein shell could significantly improve the O_2_ tolerance of encapsulated [NiFe]-hydrogenases. Interestingly, this improvement appears even greater than that of [FeFe]-hydrogenase-packaged shells.^29^ As [NiFe]-hydrogenase is relatively O_2_-tolerant compared to [FeFe]-hydrogenase, even low levels of O_2_ within the shell may substantially impede the H_2_-evolution activity of [FeFe]-hydrogenases than that of [NiFe]-hydrogenases in O_2_-tolerance assays. Hence, the higher O_2_ tolerance of [NiFe]-hydrogenase-shells than that of [FeFe]-hydrogenase-packaged shells might be due to the low O_2_ but not strictly O_2_-free microenvironment created within the carboxysome shell and the potentially different packaging efficiencies of the two hydrogenases within the shells. The detailed mechanisms underlying the catalytic improvement of encapsulated hydrogenases and their regulatory factors merit further investigation. Nevertheless, it makes this [NiFe]-hydrogenase-encapsulated catalyst a promising candidate for biohydrogen generation in applications.

Furthermore, we incubated HyaAB-Shell and free HyaAB-EP at 65 °C for 20 minutes under anaerobic conditions. Free HyaAB-EP showed a ∼67% decline in H_2_-evolution activity at pH 8 (from 32.9 ± 2.7 nmol H_2_ mg^−1^ min^−1^ to 10.8 ± 1.4 nmol H_2_ mg^−1^ min^−1^, *n* = 3) (Fig. 4A and B), indicating that high temperature could affect the hydrogenase activity of *Ec*Hyd-1, consistent with previous results.^10^ In contrast, HyaAB-Shell incubated at 65 °C preserved ∼54% of the H_2_-evolution activity (from 491.4 ± 21.0 nmol H_2_ mg^−1^ min^−1^ to 264.4 ± 12.1 nmol H_2_ mg^−1^ min^−1^ at pH 8, *n* = 3), indicating that shell encapsulation provides the physical protection to enhance the thermostability and H_2_-evolution activity of encased [NiFe]-hydrogenases.

## Conclusions

In summary, we generate genetic constructs using synthetic biology techniques to reprogramme the α-carboxysome shells as unique nanoreactors that encapsulate functional [NiFe]-hydrogenases for sustainable catalysis. The engineered hybrid biocatalyst demonstrates remarkably increased catalytic rates of hydrogen evolution under aerobic and anaerobic conditions, as well as improved oxygen tolerance and thermostability of [NiFe]-hydrogenase cargos by shell encapsulation for a longer functional lifetime. We also show an efficient encapsulation approach using the C-terminus of CsoS2 as a general EP to sequester foreign cargos within the α-carboxysome shells. Future studies will focus on further optimization of the encapsulation efficiency, cargo packaging, and structural homogeneity of the produced biocatalysts. The hydrogenase-integrating hybrid biocatalysts open new avenues for advancing our understanding of the self-assembly principles of protein organelles, and have enormous potential to recruit various cargo enzymes and multicomponent assemblies, modulate shell size and permeability, and combine inorganic/organic materials for the future development of new electrocatalytically active devices to achieve enhanced performance and robustness in industrial biocatalysis applications.

## Author contributions

Qiuyao Jiang: conceptualization, methodology, validation, formal analysis, investigation, resources, writing – original draft. Tianpei Li: methodology, formal analysis, writing – original draft. Jing Yang: methodology, investigation. Catherine M. Aitchison: methodology, investigation. Jiafeng Huang: methodology, investigation. Yu Chen: methodology, investigation. Fang Huang: formal analysis, writing – review & editing. Qiang Wang: formal analysis, writing – review & editing. Andrew I. Cooper: supervision, writing – review & editing. Lu-Ning Liu: conceptualization, formal analysis, supervision, visualization, writing – review & editing, funding acquisition.

## Conflicts of interest

A patent (WO2022074380A1) has been granted for the development and use of the α-carboxysome shell.

## Supplementary Material

TB-011-D2TB02781J-s001
