# Supplementary material for: Synthetic engineering of a new biocatalyst encapsulating [NiFe]-hydrogenases for enhanced hydrogen production
Source: J Mater Chem B. 2023 Feb 20;11(12):2684–92. doi: 10.1039/d2tb02781j (PMC10032307; doi:10.1039/d2tb02781j)
Supplement: TB-011-D2TB02781J-s001 [file TB-011-D2TB02781J-s001.pdf]

Electronic Supplementary Material (ESI) for Journal of Materials Chemistry B.
This journal is © The Royal Society of Chemistry 2023

Supplementary Information

Synthetic engineering of a new biocatalyst encapsulating [NiFe]-hydrogenases for enhanced hydrogen production

Qiuyao Jiang[a], Tianpei Li[a,b], Jing Yang[a,c], Catherine M. Aitchison[c], Jiafeng Huang[a], Yu Chen[a], Fang Huang[a], Qiang Wang[b], Andrew I. Cooper[c], and Lu-Ning Liu[a,d]*

[a] Institute of Systems, Molecular and Integrative Biology, University of Liverpool, Liverpool L69 7ZB, United Kingdom. Email: luning.liu@liverpool.ac.uk.

[b] State Key Laboratory of Crop Stress Adaptation and Improvement, School of Life Sciences, Henan University, Kaifeng 475004, China.

[c] Materials Innovation Factory and Department of Chemistry, University of Liverpool, Liverpool L7 3NY, United Kingdom.

[d] College of Marine Life Sciences, and Frontiers Science Center for Deep Ocean Multispheres and Earth System, Ocean University of China, Qingdao 266003, China.

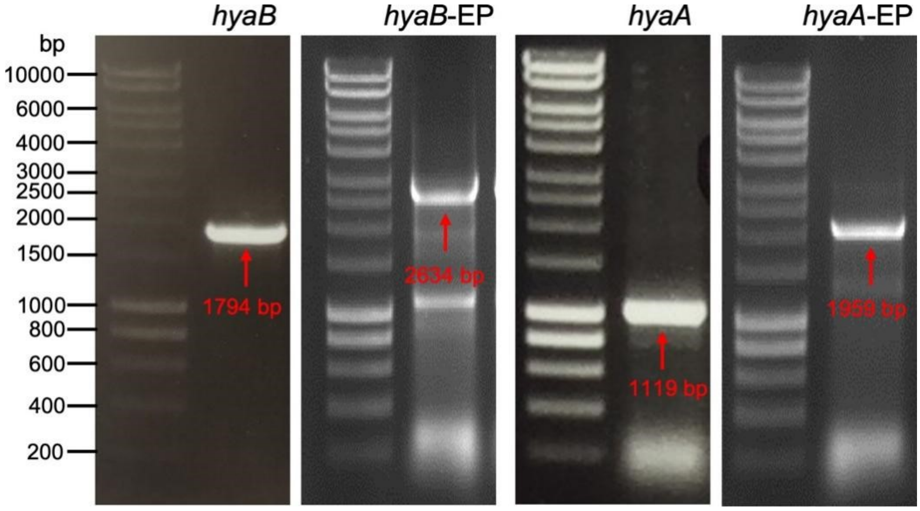

Supplementary Figure 1. Construction of the vectors expressing EcHyd-1, verified by PCR (*hyaA, hyaB, hyaA*-EP, *hyaB*-EP).

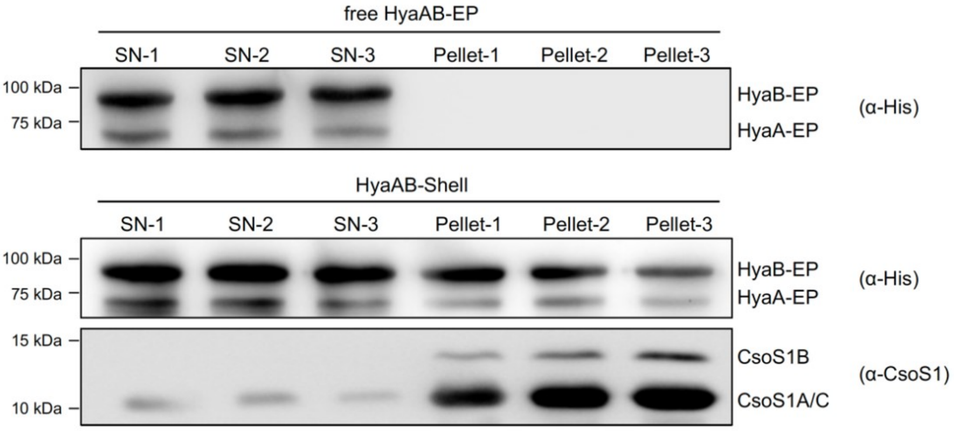

Supplementary Figure 2. Encapsulation of HyaAB with recombinant α-carboxysome shells. Immunoblot analysis of the supernatants and pellets of cell extracts after 50,000 x *g* centrifugation from *E. coli* strains producing HyaAB-EP alone or co-expressing HyaAB-EP with α-carboxysome shells (with three biological repeats), using anti-His and anti-CsoS1 antibodies, respectively.

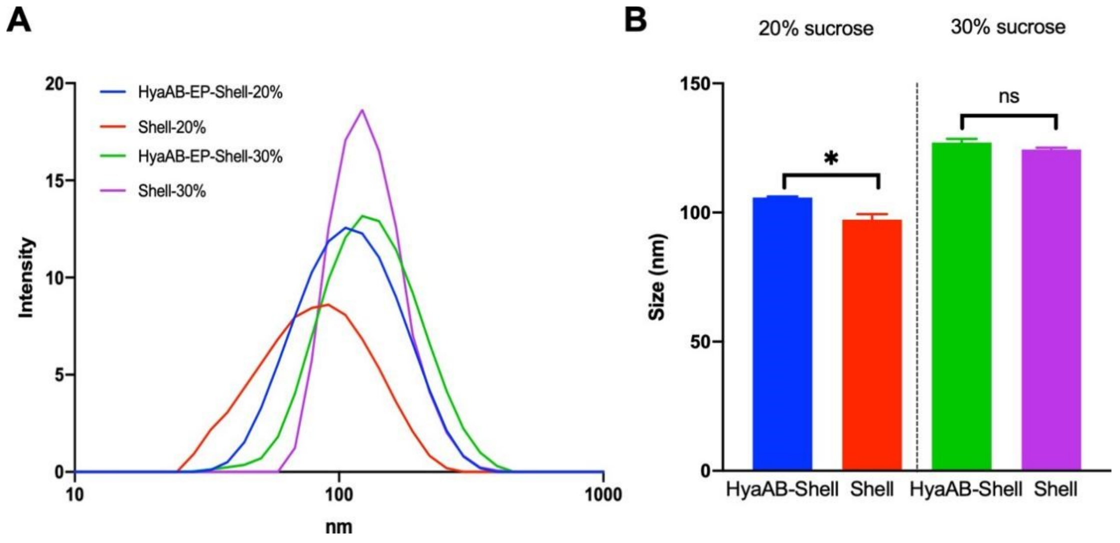

Supplementary Figure 3. Comparison of the sizes of HyaAB-Shell assemblies and empty shells. (**A**) Diameters of α-carboxysome shells and HyaAB-Shell assemblies in the 20% and 30% sucrose fractions after sucrose gradient centrifugation, measured by Dynamic Light Scattering (DLS). (**B**) Average diameters of HyaAB-Shell catalysts and empty shells in the 20% and 30% sucrose fractions measured by DLS. Values represent mean ± standard deviation, $n = 3$ biologically independent experiments. *ns*, non-significant difference. * $p = 0.02$, two-tailed unpaired t-test.

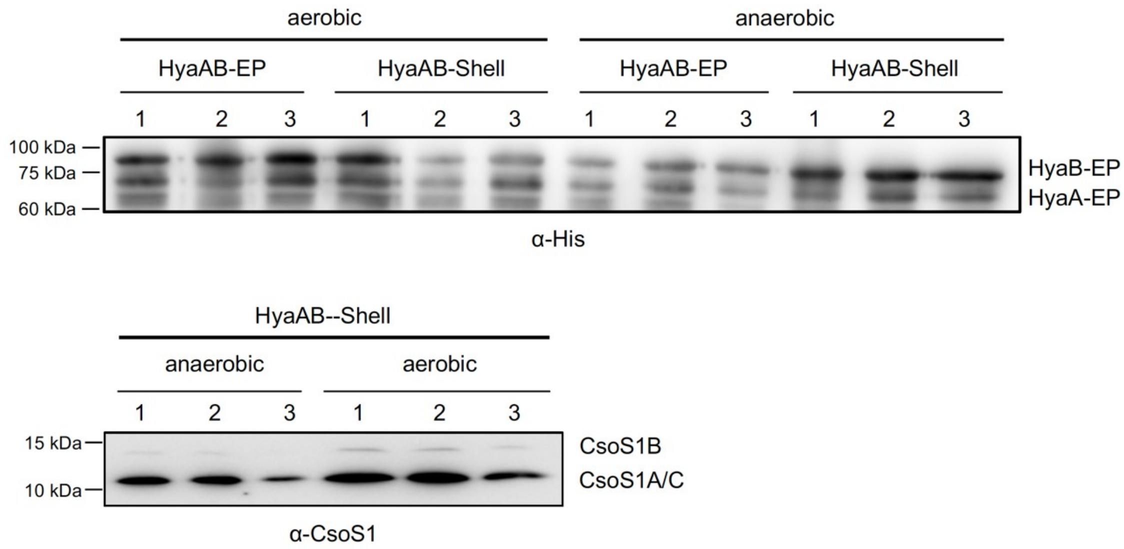

Supplementary Figure 4. Immunoblot analysis of HyaAB-Shell and unencapsulated HyaAB. Heterologously expressed *Ec*Hyd-1 and α-carboxysome shell proteins were determined using anti-6xHis-tag and anti-CsoS1A/C/B antibodies.

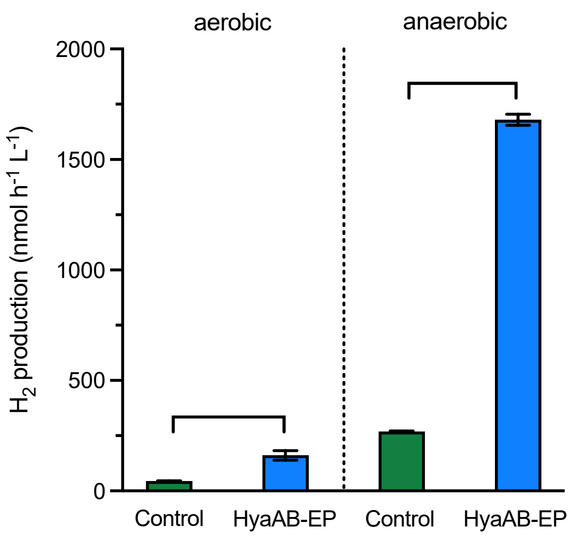

Supplementary Figure 5. ***In vivo*** **hydrogen production of wild-type** ***E. coli*** **BL21(DE3) and** ***E. coli*** **cells expressing free HyaAB-EP after 16-hour induction under aerobic (left) and anaerobic (right) conditions.** ***, p = 0.001, ****, p < 0.0001, two-tailed unpaired t-test. Error bars represent the standard deviation of the mean of three biological replicates.

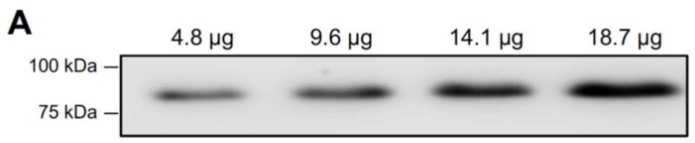

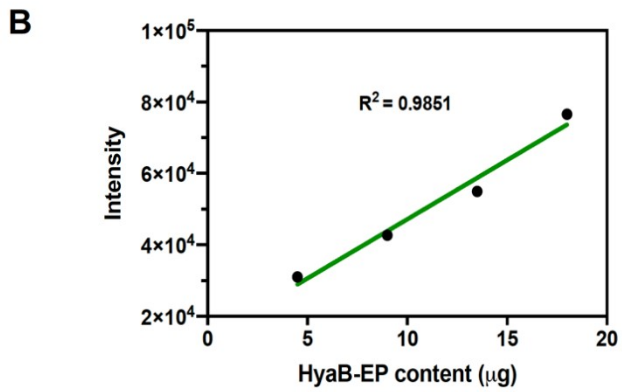

Supplementary Figure 6. A linear correlation was observed between the quantitation of HyaB-EP by BCA protein assay kit and densitometric quantitation from immunoblot analysis. (**A**) Immunoblot analysis of free HyaB-EP using an anti-His antibody. Isolated free HyaB-EP at various protein concentrations were loaded onto SDS-PAGE gels for quantification by immunoblot analysis. (**B**) The linear relationship between HyaB-EP content quantified by BCA protein assay and immunoblot analysis based on three biologically independent experiments. Densitometric quantitation of HyaB-EP levels was determined by using ImageJ.

Supplementary Table 1. Primers used in this study. Overlap sequences for Gibson Assembly are underlined.

Primers	Nucleotide sequences	Note
pCDF-HyaB-FW	CCATCACCATCATCACCACAGCCAGAGCACTCAGTACGAAACT	amplification of *hyaB* (plasmid pCDF-HyaB)
pCDF-HyaB-RV	TCGACTTAAGCATTATGCGGCCGCATTAACGCACCTGCACGGAGATCAGC	
pCDF-HyaA-FW	GTTAAGTATAAGAAGGAGATATACACATCACCATCATCACCACATGAATAACGAGGAAACA	amplification of *hyaA* (plasmid pCDF-HyaAB)
pCDF-HyaA-RV	CGCAGCAGCGGTTTCTTTACCAGACTCATGCCTGTTTATCCTC	
pCDF-HyaB-EP-FW	CCATCACCATCATCACCACAGCCAGAGCACTCAGTACGAAACT	amplification of *hyaB* (plasmid pCDF-HyaB-EP)
pCDF-HyaB-EP-RV	ACGCACCTGCACGGAGATCAGCTCG	
pCDF-HyaB-EP-FW	CGAGCTGATCTCCGTGCAGGTGCGTACGAGCACCCCAGAGCCC	amplification of *csoS2*-Cterm (plasmid pCDF-HyaB-EP)
pCDF-HyaB-EP-RV	AGCATTATGCGGCCGCAAGCTTCAACCGCGCGCGCCGCC	
pCDF-HyaA-FW	GTTAAGTATAAGAAGGAGATATACACATCACCATCATCACCACATGAATAACGAGGAAACA	amplification of *hyaA* (plasmid pCDF-HyaAB-EP)
pCDF-HyaA-RV	ACCCATTTGCGGAATATCGACCACG	
pCDF-HyaA-EP-FW	CGTGGTCGATATTCCGCAAATGGGTACGAGCACCCCAGAGCCC	amplification of *csoS2*-Cterm (plasmid pCDF-HyaB-EP)
pCDF-HyaA-EP-RV	CGCAGCAGCGGTTTCTTTACCAGACTCGATCAACCGCGCGCGCCGCC	
HyaB-seq-FW	GGATCTCGACGCTCTCCCT	Sequencing primers for pCDF-HyaB/pCDF-HyaB-EP
HyaB-seq-RV	CGATTATGCGGCCGTGTACAA	
HyaA-seq-FW	TTGTACACGGCCGCATAATC	Sequencing primers for pCDF-HyaAB/pCDF-HyaAB-EP
HyaA-seq-RV	GCTAGTTATTGCTCAGCGG	
